# Gallium-doped thermochemically treated titanium reduces osteoclastogenesis and improves osteodifferentiation

**DOI:** 10.3389/fbioe.2023.1303313

**Published:** 2023-12-07

**Authors:** David Piñera-Avellaneda, Judit Buxadera-Palomero, Maria-Pau Ginebra, Elisa Rupérez, José María Manero

**Affiliations:** ^1^ Biomaterials, Biomechanics and Tissue Engineering Group, Department of Materials Science and Engineering, Barcelona East School of Engineering (EEBE), Technical University of Catalonia (UPC), Barcelona, Spain; ^2^ Barcelona Research Center in Multiscale Science and Engineering, EEBE, Barcelona, Spain; ^3^ Institut de Recerca Sant Joan de Déu, Barcelona, Spain; ^4^ Institute for Bioengineering of Catalonia (IBEC), Barcelona, Spain

**Keywords:** titanium implant, gallium, osteoclast, osteoporosis, bone metastasis, ferroptosis

## Abstract

Excessive bone resorption is one of the main causes of bone homeostasis alterations, resulting in an imbalance in the natural remodeling cycle. This imbalance can cause diseases such as osteoporosis, or it can be exacerbated in bone cancer processes. In such cases, there is an increased risk of fractures requiring a prosthesis. In the present study, a titanium implant subjected to gallium (Ga)-doped thermochemical treatment was evaluated as a strategy to reduce bone resorption and improve osteodifferentiation. The suitability of the material to reduce bone resorption was proven by inducing macrophages (RAW 264.7) to differentiate to osteoclasts on Ga-containing surfaces. In addition, the behavior of human mesenchymal stem cells (hMSCs) was studied in terms of cell adhesion, morphology, proliferation, and differentiation. The results proved that the Ga-containing calcium titanate layer is capable of inhibiting osteoclastogenesis, hypothetically by inducing ferroptosis. Furthermore, Ga-containing surfaces promote the differentiation of hMSCs into osteoblasts. Therefore, Ga-containing calcium titanate may be a promising strategy for patients with fractures resulting from an excessive bone resorption disease.

## Graphical Abstract

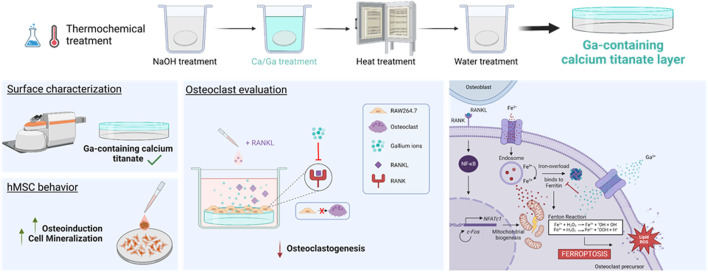

## 1 Introduction

The bone remodeling cycle is the process by which an old bone is replaced by a new bone and is controlled by cooperative work between osteocytes, osteoblasts, and osteoclasts (Szulc, 2018). Therefore, bone cells cooperate together in order to maintain a balance between bone formation and bone resorption. This balance can be altered in some diseases such as osteoporosis or bone metastasis, mainly due to an abnormal increase in osteoclast activity (Teitelbaum, 2007; Datta et al., 2008; Kim et al., 2018). The abnormally high osteoclast activity leads to a higher bone resorption and, consequently, a bone mass loss. In osteoporosis, the increase in osteoclast activity is mainly due to aging or menopause, while in the case of bone cancer and metastasis, the bone remodeling cycle becomes a vicious cycle in which the cancer cells induce a higher osteoclast activity which destroys the bone matrix allowing the spread of the tumor (Maurizi and Rucci, 2018; Coleman et al., 2020). Treatments used currently, such as bisphosphonates or denosumab, focus on reducing osteoclast-related bone resorption by inhibiting the receptor activator of the nuclear factor κ-B ligand (RANKL) (Deeks, 2018; Deardorff et al., 2022). However, even with the use of anti-resorptive agents, the risk of fractures may remain high and even require the use of prosthesis (Langdahl, 2021).

Titanium implants are the most used biomaterials to repair the damaged bone (Geetha et al., 2009; Kaur and Singh, 2019; Vidal et al., 2020). Despite their suitability, achieving adequate osteointegration in an adverse environment is an arduous labor, so the combination of the traditional treatment with an implant capable of reducing the abnormal bone resorption in the injured bone would be the ideal approach (Langdahl, 2021). In this manner, the implant will not only substitute the injured bone but also prevent the bone resorption caused by the disease. The use of gallium (Ga) has been proposed as a modification of titanium implants with this aim (Yamaguchi et al., 2017). This semi-metal has been demonstrated to inhibit bone resorption in diseases such as osteoporosis, bone metastasis, and Paget’s disease (Chitambar, 2010; Bonchi et al., 2014). Moreover, Ga(NO_3_)_3_ was approved by the US Food and Drug Administration (FDA) to be used for treating malignancy-associated hypercalcemia (Minandri et al., 2014). It has been suggested that Ga may avoid bone resorption by inhibiting osteoclast recruitment, thereby reducing the expression of the nuclear factor of activated T cells (nFATc1) (Verron et al., 2012a; Verron et al., 2012b), the main transcription factor involved in osteoclastogenesis (Gohda et al., 2005; Takayanagi, 2007).

Despite its prominent role in osteoclast differentiation, few studies have deepened on elucidating the mechanism by which Ga may reduce bone resorption. Ga has been defined as a “Trojan Horse” due to its similarity to iron (Fe) (Valappil et al., 2008; Goss et al., 2018). In its cationic form (Ga^3+^), this semi-metal presents an ionic radius almost identical to that of a ferric ion (Fe^3+^) (Bonchi et al., 2014). Therefore, Ga^3+^ could also bind to transferrin (Tf) in the bloodstream and be taken up by cells via Tf receptor 1 (TfR1) (Chikh et al., 2007; Chitambar, 2016). Once inside, Ga^3+^ could intervene in the iron-dependent metabolic pathways and disrupt the involved processes. In fact, this is the mechanism by which Ga exerts antibacterial activity (Li et al., 2022). Moreover, it has recently been related osteoclastogenesis with a novel kind of iron-dependent cell death: ferroptosis (Gao et al., 2022; Liu et al., 2022).

In a previous study, we developed a Ga coating on porous three-dimensional (3D) printed titanium by modifying the surface with a thermochemical treatment using Ga(NO_3_)_3_ (Rodríguez-Contreras et al., 2020). This modification created a Ga-containing calcium titanate layer on the surface that showed an improved bioactivity, antibacterial activity, and increased mineralization capacity of osteoblast-like cells (SaOS-2). The objective of this work is to prove the capacity of the coating of inhibiting osteoclastogenesis and promoting osteodifferentiation. To achieve the objective, the RAW 264.7 macrophage cell line was used to assess multinuclearity, TRAP staining, and osteoclastic gene evaluation. After that, the involvement of Ga in the osteodifferentiation was evaluated by immunofluorescence, proliferation, ALP activity, mineralization, and osteogenic profile by using human mesenchymal stem cells (hMSCs) on the material.

## 2 Materials and methods

### 2.1 Materials

Commercially pure Ti grade 2 solid discs of 10 mm diameter and 2 mm thickness were used for *in vitro* characterization. Ti discs were polished with SiC grinding papers (P800 and P1200) and, subsequently, a colloidal silica suspension of 0.05 μm. Before their use, Ti samples were ultrasonically cleaned with acetone, 2-propanol, and distilled water twice for 5 min each and finally dried with N_2_ and stored.

### 2.2 Thermochemical treatment

The thermochemical treatment was carried out by following the procedure published by Rodríguez-Contreras et al. (2020), and it is shown in Table 1. In the first step, the samples were subjected to NaOH attack to create sodium hydrogen titanate. Next, the samples were washed and soaked in a calcium (Ca) solution mixed with three different concentrations of Ga solution (Ga5: 5 mM Ga(NO_3_)_3_, Ga10: 10 mM Ga(NO_3_)_3_, and Ga25: 25 mM Ga(NO_3_)_3_) to allow Ga incorporation. Moreover, there were samples only subjected to the thermochemical treatment without the addition of Ga for comparing results. Then, the samples were heated to 600°C. Finally, they were soaked in water, dried with N_2_, and stored under low-humidity conditions.

**TABLE 1:** Stages of thermochemical treatments performed and sample references.

Sample	first step	second step	third step	fourth step
Ti–Ca	5M NaOH	100-mM C₄H₆CaO₄	heat treatment	H_2_O treatment
	24h/60°C	24h/40°C	600°C/1h	24h/80°C
Ga5	5M NaOH	100-mM CaCl_2_	heat treatment	H_2_O treatment
		+5-mM Ga(NO_3_)_3_		
	24h/60°C	24h/40°C	600°C/1h	24h/80°C
Ga10	5M NaOH	100-mM CaCl_2_	heat treatment	H_2_O treatment
		+10-mM Ga(NO_3_)_3_		
	24h/60°C	24h/40°C	600°C/1h	24h/80°C
Ga25	5M NaOH	100-mM CaCl_2_	heat treatment	H_2_O treatment
		+25-mM Ga(NO_3_)_3_		
	24h/60°C	24h/40°C	600°C/1h	24h/80°C

### 2.3 Surface characterization

#### 2.3.1 Fourier transform confocal laser Raman spectrometry

The treated samples were characterized by using an inVia™ Qontor^®^ confocal Raman microscope (Renishaw Centrus 2957T2, Gloucestershire, United Kingdom). A regular mode laser with a wavelength of 532 nm and a grating of 2400 L/mm was used. Spectra were acquired with an objective of 50 magnifications, 1 s of exposure time, and 40 accumulations.

#### 2.3.2 Ion release

Ga^3+^ from the treated samples was evaluated according to the ISO-10993-12 standard by inductively coupled plasma mass spectrometry (ICP-MS, Agilent 5100 SVD ICP-OES, CA, United States). Each sample was immersed in Dulbecco’s modified Eagle medium (DMEM) (1 mL of DMEM per gram of sample), and after each timepoint, 1 mL of the suspension was taken, filtered, and diluted 1:10 in 2% nitric acid. The same volume was replaced with a fresh medium.

### 2.4 *In vitro* cell evaluation

#### 2.4.1 Cell culture

Mouse monocyte macrophage RAW 264.7 cells (ATCC, United States) were cultured in DMEM supplemented with 10% FBS, 1% penicillin/streptomycin (50 U/mL and 50 μg/mL, respectively), and 20-mM N-(2-hydroxyethyl)piperazine-N′-ethanesulfonic acid buffer solution, all from Gibco^TM^. Human bone marrow mesenchymal stem cells (ATCC, United States) were cultured in advanced Dulbecco’s modified Eagle medium with D-glucose, nonessential amino acids, and sodium pyruvate and supplemented with 10% fetal bovine serum (FBS), 1% penicillin/streptomycin (50 U/mL and 50 μg/mL, respectively), and 20-mM N-(2-hydroxyethyl)piperazine-N′-ethanesulfonic acid buffer solution, all from Gibco^TM^. All cells were maintained and expanded at 37°C in a 95% humidified atmosphere containing 5% of CO_2_. Cell density and passage are indicated in each particular experiment.

#### 2.4.2 Sample sterilization and incubation

Solid discs of Ti, both treated and untreated (control), were sterilized by washing them in ethanol (70%, v/v) for 20 min and then rinsed thrice with PBS.

#### 2.4.3 Multinuclearity study of RAW 264.7 cells

RAW 264.7 cells were seeded on Ti surfaces at 10,000 cells per sample. The medium used for the seeding was supplemented with RANKL (60 ng/mL) and maintained in culture for 4 and 6 days, changing the medium once and thrice, respectively. After incubation, the cell medium was removed, and the samples were rinsed with 500-μL PBS; then, the cells were fixed with 500 μL (4%) of paraformaldehyde (Sigma) for 20 min at RT. After fixation, the samples were washed thrice with PBS-Gly (20 mM glycine in PBS) for 5 min. Then, the cells were permeabilized with 0.05% Triton X-100 in PBS for 20 min at RT, rinsed thrice again with PBS-Gly, and incubated with Alexa Fluor 546 Phalloidin–Rodhamine (A22283, Invitrogen, United States) (1:400 in PBS-0.05% triton) during 1 h in dark. The samples were washed thrice with PBS-Gly, and DAPI (1:1000 in PBS-Gly) was added for 2 min in the dark. After incubation, the samples were finally rinsed with PBS-Gly and ready for image analysis. Randomized images of each sample were captured using Zeiss LSM 800 (Zeiss, Germany).

#### 2.4.4 Tartrate-resistant acid phosphatase staining

RAW 264.7 cells were seeded on Ti surfaces at 10,000 cells per sample. The medium used for the seeding was supplemented with RANKL (60 ng/mL) and maintained in culture for 6 days, changing the medium thrice. After incubation time, the medium was removed and rinsed with PBS. Then, the samples were stained using the Leukocyte Acid Phosphatase (TRAP) kit (Sigma-Aldrich, United States) following the provided procedure. After 1 h of incubation, the samples were thoroughly rinsed with deionized water, and then, hematoxylin solution was added for staining the nuclei. Then, the samples were rinsed in alkaline tap water, dried, and evaluated using an Olympus BX51-P bright-field microscope (Olympus Corp., Japan)

#### 2.4.5 RAW 264.7 osteoclastic expression

RAW 264.7 cells were seeded on Ti surfaces at 10,000 cells per sample. The medium used for the seeding was supplemented with RANKL (60 ng/mL) and maintained in culture for 4 days, changing the medium twice. RNA extraction and RT–PCR were carried out following the procedure explained above. GAPDH was used as a housekeeping gene, and the relative gene expression levels were evaluated using the 2^ΔΔ−Ct^ method. Primer sequences used are shown in Table 2 **.**

**TABLE 2:** Osteoclastic primer sequences used for RT–PCR.

Gene	forward sequence (5′ to 3′)	reverse sequence (5′ to 3′)
GAPDH	TGT​GTC​CGT​CGT​GGA​TCT​GA	TTG​CTG​TTG​AAG​TCG​CAG​GAG
NFATc1	GGT​AAC​TCT​GTC​TTT​CTA​ACC​TTA​AGC​TC	GTG​ATG​ACC​CCA​GCA​TGC​ACC​AGT​CAC​AG
TRAP	TAC​CTG​TGT​GGA​CAT​GAC​C	CAG​ATC​CAT​AGT​GAA​ACC​GC
c-Fos	CCA​AGC​GGA​GAC​AGA​TCA​ACT​T	TCC​AGT​TTT​TCC​TTC​TCT​TTC​AGC​AGA​T
MMP9	TCC​AGT​ACC​AAG​ACA​AAG​CCT​A	TTGCACTGCACGGTTGAA

#### 2.4.6 hMSC adhesion and morphology assay

hMSCs at passage 4 were seeded on Ti surfaces at 15,000 cells per sample and incubated for 24 h at 37°C. After incubation, the cell medium was removed and the samples were rinsed with 500-μL PBS; then, the cells were fixed with 500 μL (4%) of paraformaldehyde (Sigma) for 20 min at RT. After fixation, the samples were washed thrice with PBS-Gly (20-mM glycine in PBS) for 5 min. Then, the cells were permeabilized with 0.05% Triton X-100 in PBS for 20 min at RT, rinsed thrice again with PBS-Gly, and blocked with 1% BSA in PBS for 30 min. Then, the medium was removed, and the cells were incubated with the primary antibody mouse anti-vinculin (V9131, Sigma-Aldrich, United States) (1:400 in 1% BSA in PBS). After incubation, the samples were washed thrice with PBS-Gly and incubated with secondary antibody Alexa Fluor 488 goat anti-mouse IgG (R37120, Invitrogen, United States) for 1 h in the dark, following the manufacturer’s protocol. After that, the samples were washed with PBS-Gly and incubated with Alexa Fluor 546 Phalloidin–Rodhamine (A22283, Invitrogen, United States) (1:400 in PBS-0.05% triton) for 1 h in the dark. The samples were washed thrice with PBS-Gly, and DAPI (1:1000 in PBS-Gly) was added for 2 min in the dark. After incubation, the samples were finally rinsed with PBS-Gly and ready for image analysis. Randomized images of each sample were captured using Zeiss LSM 800 (Zeiss, Germany) and analyzed with Fiji/ImageJ (Schindelin et al., 2012).

#### 2.4.7 hMSC proliferation assay

hMSCs at passage 4 were seeded on Ti surfaces at 15,000 cells per sample and maintained in culture for 1, 7, 14, and 21 days, changing the medium twice or thrice a week. After each incubation period, the cells were rinsed thrice in 500-μL PBS and incubated with 350-μL 10% of PrestoBlue^TM^ (Invitrogen, United States) for 1 h at 37°C. As a negative control, PrestoBlue^TM^ was incubated without cells. Then, 100 μL of the medium was transferred to a black 96-well plate to measure an excitation wavelength at 560 nm and an emission wavelength at 590 nm by using a Synergy HTX multimode reader (Bio-Tek, United States).

#### 2.4.8 hMSC alkaline phosphatase activity

The same cells used for the proliferation assay were employed to quantify the ALP activity on days 14 and 21. The cells were rinsed with 500-μL PBS to remove the remaining PrestoBlue^TM^ and then lysed with 500 μL of the mammalian protein extraction reagent (M-PER, Thermo Fisher Scientific, United States). The samples were incubated for 1 h at 37°C using the SensoLyte pNPP Alkaline Phosphatase Assay Kit (AnaSpec Inc., United States), and absorbance was registered at 405 nm using a Synergy HTX multimode reader (Bio-Tek, United States). The resulting ALP quantity was normalized *versus* Ti–Ca and their corresponding cell numbers obtained in the cell proliferation assay.

#### 2.4.9 hMSC mineralization assay

hMSCs were seeded at passage 4 on Ti surfaces at 20,000 cells per sample for 21 days. After each incubation time, the cell medium was removed, and the samples were rinsed with 500-μL PBS; then, the cells were fixed with 500 μL (4%) of paraformaldehyde (Sigma) for 20 min at RT. After that, the samples were rinsed twice with Mili-Q water, and calcium deposits were stained with 500 µL/sample of 40-mM Alizarin Red S (Sigma-Aldrich) for 20 min with gentle shaking. Unincorporated dye was removed and washed with Mili-Q water until the medium became transparent. Then, six randomized images of each sample were captured using an Olympus BX51-P bright-field microscope (Olympus Corp., Japan). Quantification of calcium deposits was performed with Fiji/ImageJ (Schindelin et al., 2012).

#### 2.4.10 hMSC osteogenic expression

MSCs were seeded at passage 3 on Ti surfaces at 30,000 cells per sample for 3 and 7 days in order to evaluate the gene expression. After each time point, the cells were unattached and pellets were collected. Then, the cells were lysed and total RNA was extracted and purified using the RNeasy Mini Kit columns (Qiagen, Hilden, Germany) as described in the manufacturer’s instructions. Then, cDNA synthesis was performed using the Maxima First Strand cDNA Synthesis Kit for qRT–PCR, with dsDNase (Thermo Fisher Scientific, #K1671). RT–PCR was carried out on a Mic real-time PCR cycler (Bio Molecular Systems, Australia), and gene expression was assessed by using the QuantiNova Fast SYBR™ Green PCR Master Mix (Qiagen). β-Actin was used as a housekeeping gene, and the relative gene expression levels were evaluated using the 2^ΔΔ−Ct^ method. Primer sequences used are shown in Table 3.

**TABLE 3:** Osteogenic primer sequences used for RT–PCR.

Gene	forward sequence (5′ to 3′)	reverse sequence (5′ to 3′)
β-Actin	TTG​CCA​TCA​ATG​ACC​CCT​TCA	CGCCCCACTTGATTTTGG A
RUNX2	AAA​TGC​CTC​CGC​TGT​TAT​GAA	GCTCCGGCCCACAAATCT
COL1A1	AGG​TCC​CCC​TGG​AAA​GAA	AATCCTCGAGCACCCTGA
ALP	ATC​TTT​GGT​CTG​GCT​CCC​ATG	TTT​CCC​GTT​CAC​CGT​CCA​C
OPN	AGC​TGG​ATG​ACC​AGA​GTG​CT	TGA​AAT​TCA​TGG​CTG​TGG​AA

### 2.5 Statistical analysis

The statistical analysis was carried out by Minitab 19 software (Minitab, EE.UU), which was used to analyze whether statistically significant differences exist among the results or not. All samples were previously analyzed to determine if they followed a normal distribution by using the normality test. Then, the ANOVA test or Kruskal–Wallis test were run to work out its *p*-value, depending on if variables were normally distributed or not, respectively. Triplicates of each sample were used (n = 3). # represents statistically significant differences between treated samples and Ti. & represents statistically significant differences between treated samples and Ti–Ca for each time point (*p*-value <0.05).

## 3 Results

### 3.1 Gallium is incorporated into the titanium surface

**FIGURE 1:**
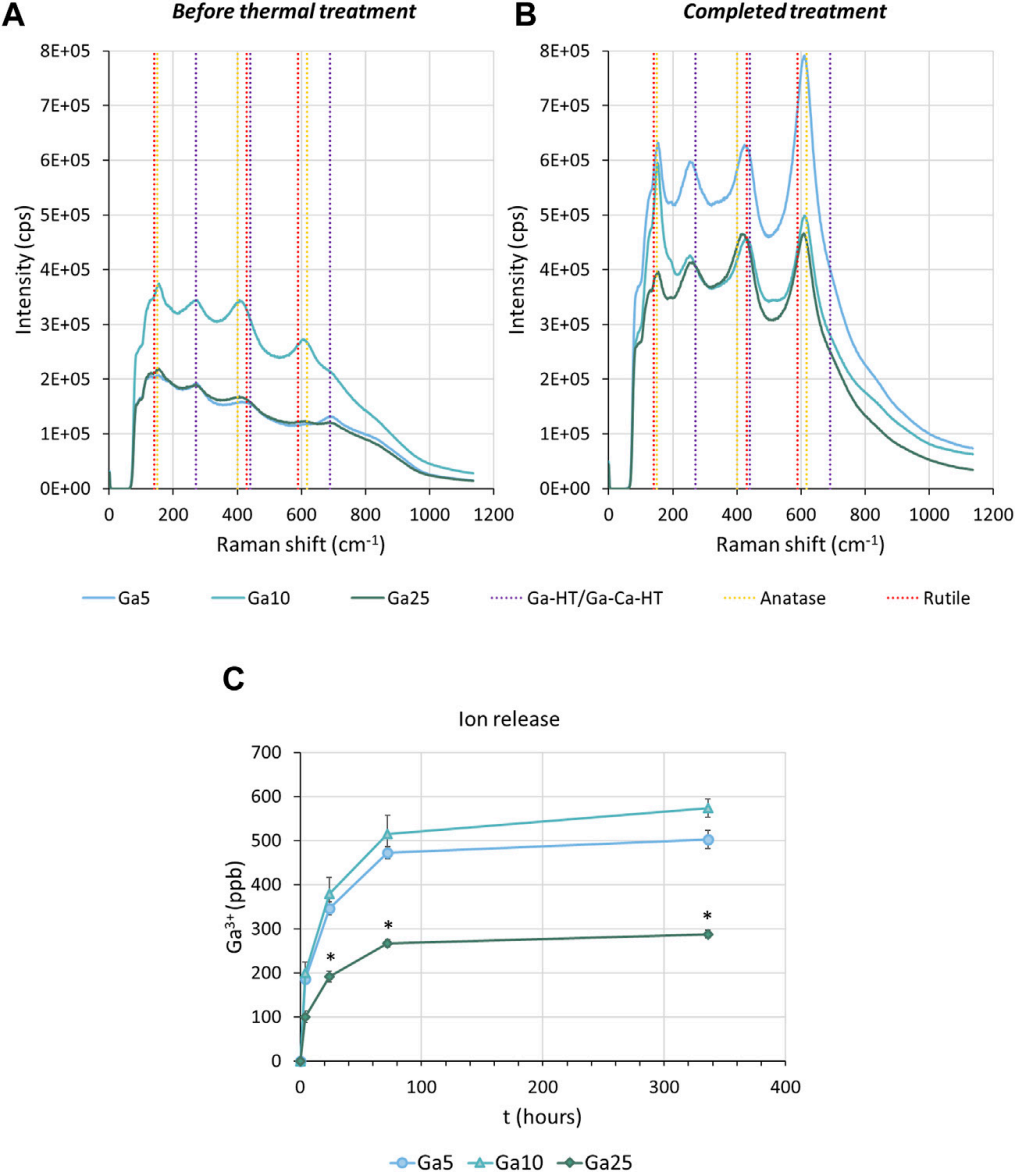
Surface characterization. **(A)** Raman spectra of Ga-containing samples before the thermal treatment. **(B)** Raman spectra of Ga-containing samples subjected to the thermochemical treatment completed. Ga-HT/Ga-Ca-HT corresponds to either gallium hydrogen titanate or gallium-containing calcium titanate. Ga-T/Ga-Ca-T corresponds to either gallium titanate or gallium-containing calcium titanate. **(C)** Accumulative curves of Ga3+ released from the surface of the Ga-containing samples. * represents statistically significant differences between samples.

Chemical characterization by Raman spectroscopy of the sample before the thermal treatment showed the characteristic bands of anatase at 400 cm^−1^, 150 cm^−1^, and 600 cm^−1^. The last two bands can also be assigned to rutile. Moreover, the bands corresponding to either gallium hydrogen titanate or gallium-containing calcium hydrogen titanate at 290 cm^−1^ and 690 cm^−1^ were also found (Figure 1A). After thermal treatment (Figure 1B), the peaks corresponding to anatase and rutile were still observed. However, a shift in the peak at 290 cm^−1^–270 cm^−1^ indicated the formation of gallium titanate. With the completed treatment, a peak at 440 cm^−1^ was found, corresponding to both gallium titanate and gallium-containing calcium titanate.

The Ga-containing samples released Ga^3+^, especially fast in the first hours (Figure 1C), as observed by ICP-MS. After 336 h (2 weeks), Ga5 had released 500.26 ppb, Ga10 570.26 ppb, and Ga25 280.69 ppb. Therefore, a slower Ga^3+^ release in Ga25 was found.

### 3.2 Ga-containing surfaces reduce osteoclast formation

**FIGURE 2:**
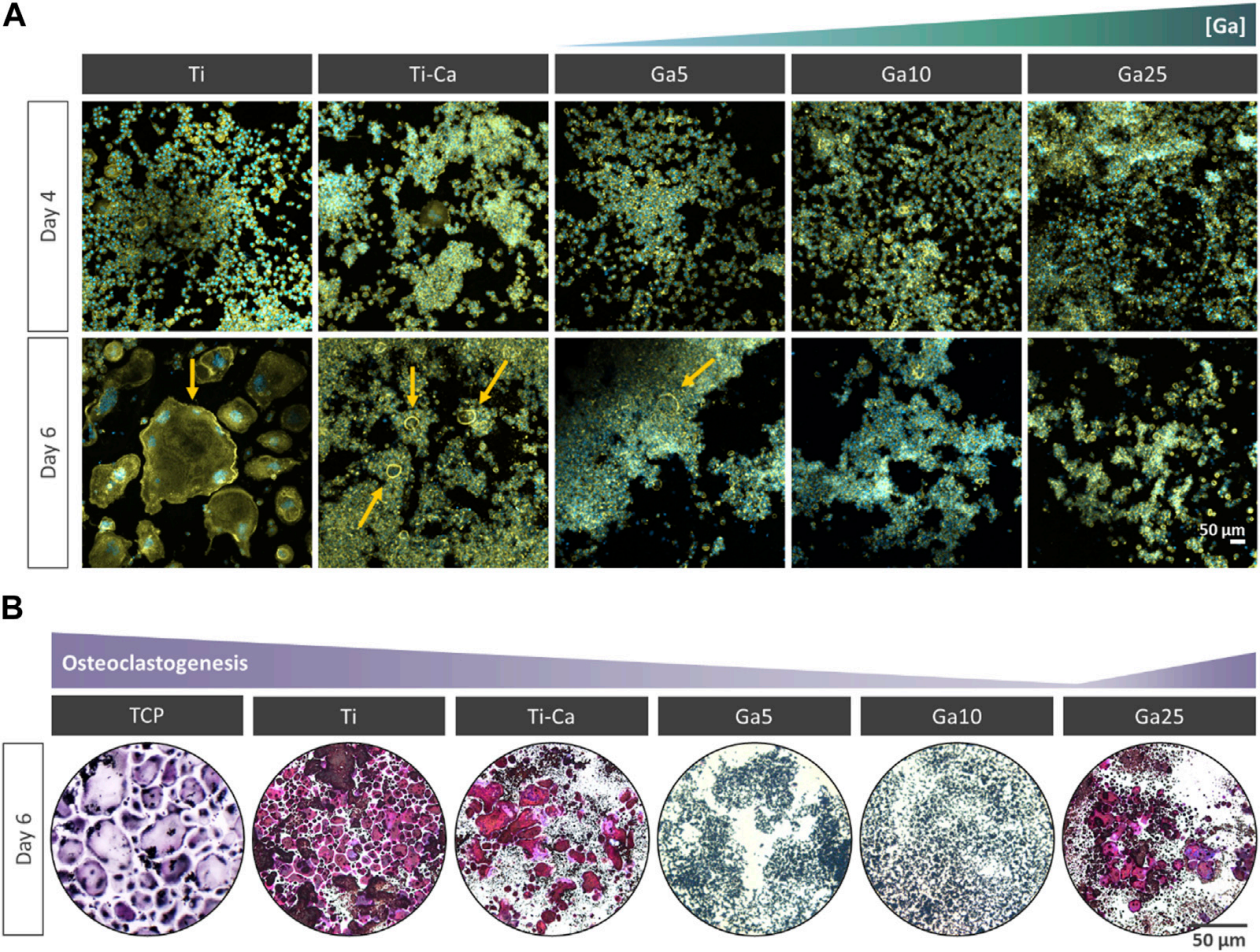
Impact of Ga incorporation into calcium titanate in osteoclastogenesis. **(A)** Representative images of RANKL-treated RAW 264.7 cells after for 4 and 6 days of culture. The cells were labeled with DAPI (nuclei, blue) and phalloidin (F-actin, green). Yellow arrow indicates multinucleated cells. **(B)** Representative images of RANKL-treated RAW 264.7 cells after 6 days and stained with TRAP staining. TRAP-positive cells are in violet.

Osteoclast formation was studied by culturing RAW 264.7 cells with the RANKL-treated medium (Figure 2A). On day 4, some small multinucleated cells were found on Ti and Ti–Ca, indicating the beginning of the osteoclastogenesis, while no evidence of multinucleated cells was found on Ga-containing surfaces. On day 6, giant multinucleated cells were clearly found on Ti, which can be identified as mature osteoclasts. On Ti–Ca, the number of multinucleated cells was higher. In the case of Ga-containing surfaces, only on Ga5 appeared shreds of evidence of multinucleated cells under the colonies formed by non-differentiated macrophages, but Ga10 and Ga25 showed no presence of multinucleated cells. In order to further investigate the results, the TRAP staining was carried out (Figure 2B). TRAP-positive cells were found on the surface of tissue culture plastic (TCP), Ti, Ti–Ca, and in lower quantity on Ga25.

### 3.3 The incorporation of Ga reduces the osteoclastic gene expression

**FIGURE 3:**
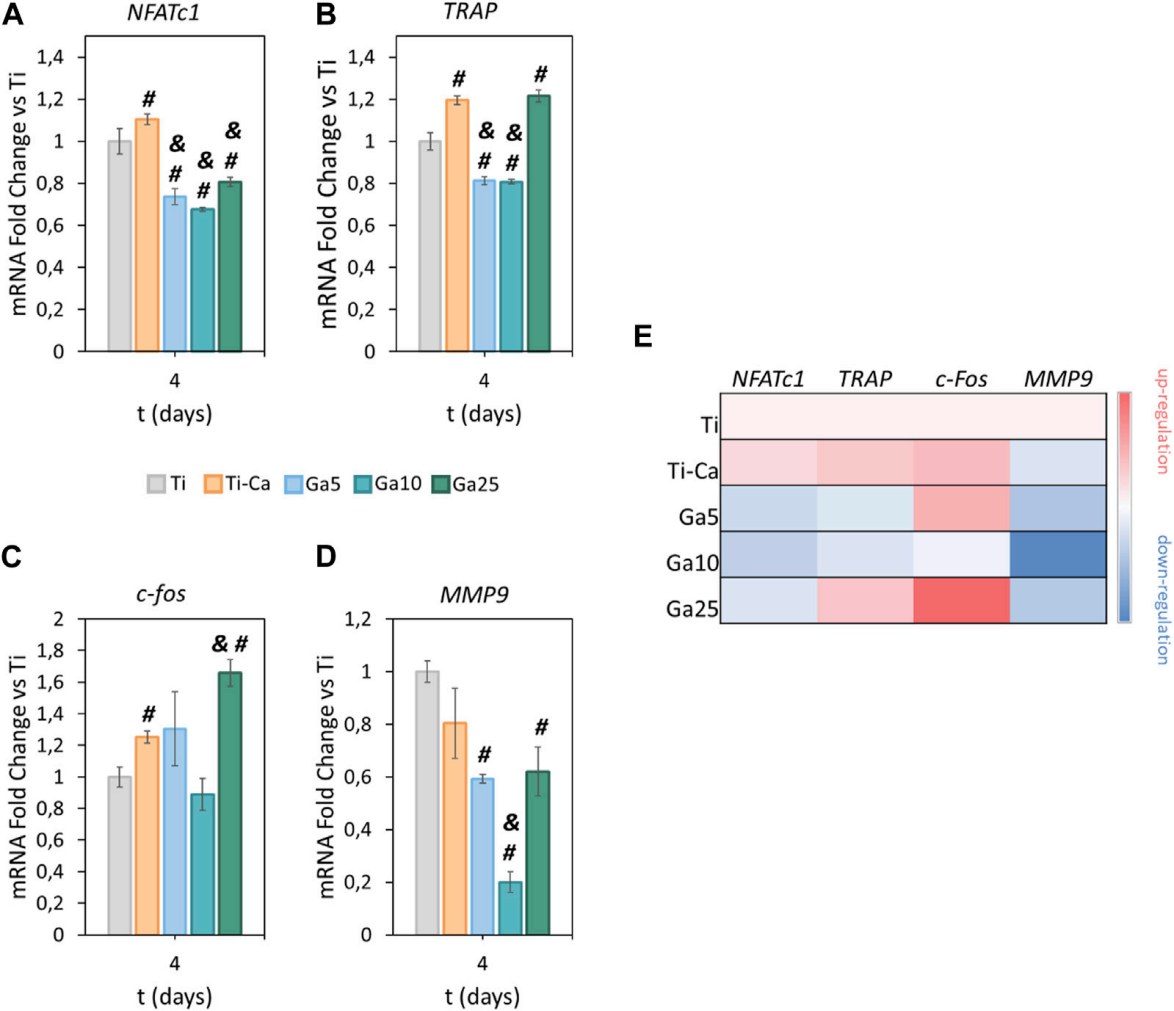
Impact of Ga-containing surfaces in osteoclastogenic gene expression. RT–PCR analyses of **(A)** NFATc1, **(B)** TRAP, **(C)** c-fos, and **(D)** MMP9 for RANKL-treated RAW 264.7 cells after 4 days. **(E)** Heatmap of osteoclastic gene expression (red = upregulation, white = no changes, and blue = downregulation). The results were normalized using GAPDH as housekeeping and represented as relative fold change to Ti. # represents statistically significant differences between treated samples and Ti. & represents statistically significant differences between treated samples and Ti–Ca for each time point (*p*-value <0.05).

NFATc1 (Figure 3A) was slightly upregulated by Ti–Ca with respect to Ti. However, Ga surfaces reduced its expression. Ga5 downregulated NFATc1 with respect to Ti by 26.4%, Ga10 by 32.4%, and Ga25 by 19.4%. With respect to Ti–Ca, the downregulation was 33.3%, 38.7%, and 27%, respectively. Concerning TRAP (Figure 3B), its expression was slightly upregulated in Ti–Ca and Ga25. In contrast, Ga5 and Ga10 reduced its expression with respect to Ti by 18.8% and 19.3%, respectively. With respect to Ti–Ca, the downregulation was 32.1% and 32.5%, respectively. Regarding c-fos (Figure 3C), only Ti–Ca and Ga25 upregulated its expression. No significant differences were found between Ti, Ga5, and Ga10. About matrix metalloproteinase 9 (MMP9) (Figure 3D), its expression was strongly downregulated on Ga-containing surfaces, especially by 80% in Ga10. Overall, Ga10 was the condition that showed a stronger downregulation of osteoclastogenic expression (Figure 3E).

### 3.4 hMSC shows adequate spreading, morphology, and proliferation on Ga-containing surfaces

**FIGURE 4:**
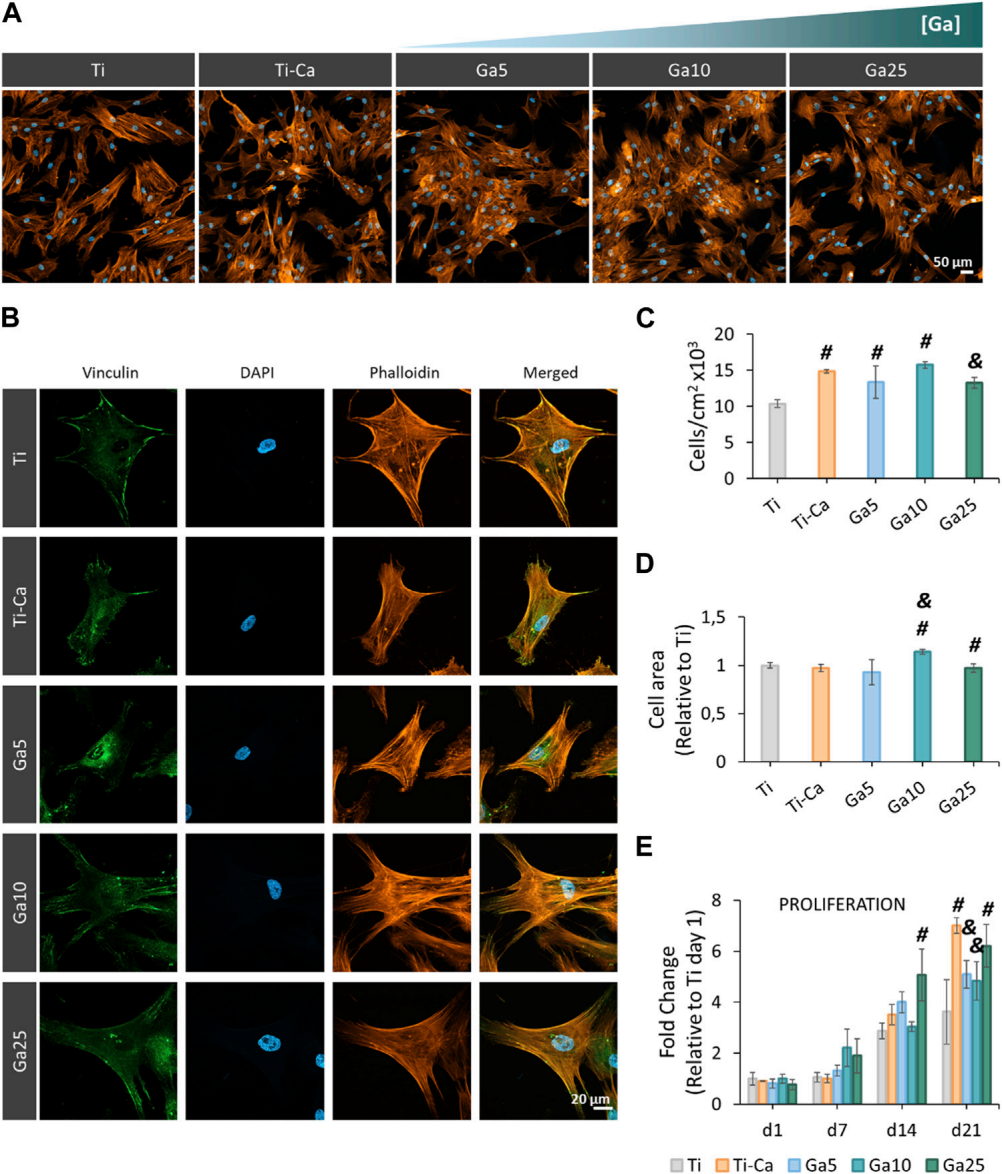
Adhesion, morphology, and proliferation of hMSCs seeded on titanium samples. **(A)** Representative images of hMSCs seeded for 24 h by using fluorescence staining. The cells were labeled with DAPI (nuclei, blue) and phalloidin (F-actin, orange). **(B)** hMSC morphology seeded on titanium samples at 24 h. The cells were labeled with DAPI (nuclei, blue), phalloidin (F-actin, orange), and vinculin (focal adhesions, green). **(C)** Quantification of the number of adhered cells per cm^2^ seeded on titanium surfaces for 24 h. **(D)** Cell area after seeding the cells for 24 h. **(E)** Proliferation of hMSCs after 21 days (d1 = day 1, d7 = day 7, d14 = day 14, and d21 = day 21). Six pictures per sample were used per quantify (n = 3). # represents statistically significant differences between treated samples and Ti. & represents statistically significant differences between treated samples and Ti–Ca for each time point (*p*-value <0.05).

hMSC seeded on the surfaces showed an adequate spreading (Figures 4A,B) and morphology, and the incorporation of Ga did not affect the typical structure of the hMSCs. Moreover, focal adhesions were found under all conditions. Ti–Ca, Ga5, and Ga10 showed a higher number of cells than Ti (Figure 4C). However, no statistically significant differences were found between Ga25 and Ti, in addition to showing a lower number of adhered cells than Ti–Ca. The cell area was similar on all surfaces (Figure 4D), but in the case of Ga10, it was improved by 13.8% with respect to Ti. The cell area in Ga25 was slightly reduced compared to Ti. Regarding cell proliferation (Figure 4E), Ti–Ca and Ga25 showed the greatest proliferation fold change with respect to Ti on day 21, and in the case of Ga5 and Ga10, proliferation was slightly lower.

### 3.5 Ga-containing surfaces improve ALP activity and mineralization

**FIGURE 5:**
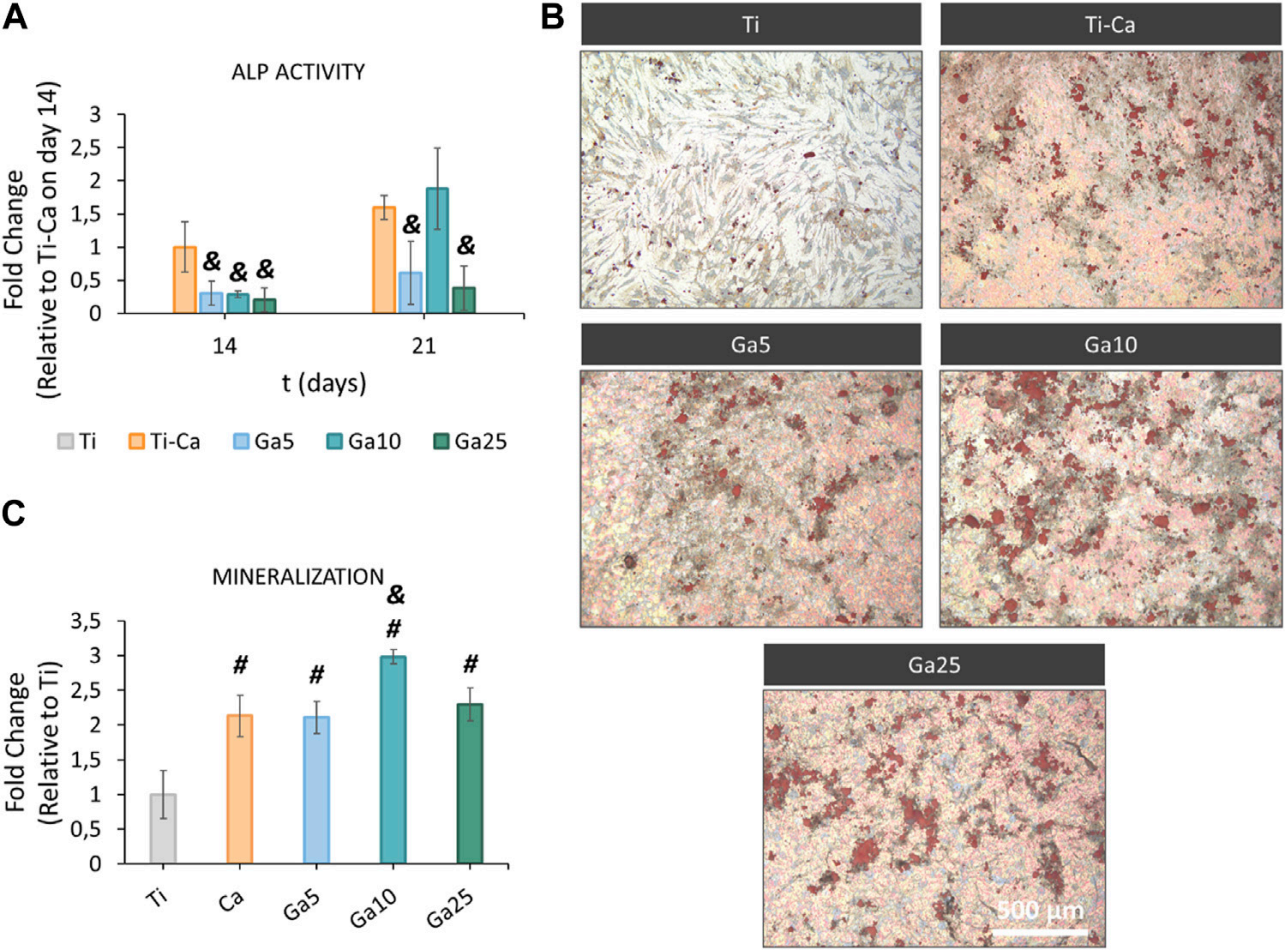
ALP activity and mineralization of hMSCs on titanium samples. **(A)** ALP activity of hMSCs on days 14 and 21. Results expressed as fold change to Ti–Ca on day 14. **(B)** Representative images of calcium deposits produced by hMSCs on day 21. Calcium deposits were labeled in red using Alizarin red staining. **(C)** Quantification of the calcium deposits. Results were expressed as fold change to Ti. Each condition was replicated in triplets (n = 3), and six pictures per sample were used per quantify. # represents statistically significant differences between treated samples and Ti. & represents statistically significant differences between treated samples and Ti–Ca for each time point (*p*-value <0.05).

ALP activity (Figure 5A) was found in Ti–Ca, Ga5, Ga10, and Ga25 on day 14. In this case, the activity of this osteoinductive marker in all Ga-containing conditions was lower than that in Ti–Ca. On day 21, the activity was also lower in Ga5 and Ga25, but in Ga10, it was the same as that in Ti–Ca. Ti did not show ALP activity. On one hand, Ga surfaces failed to enhance ALP activity much; on the other hand, they were able to improve mineralization (Figure 5B). Ti–Ca and all Ga-containing conditions stimulated the production of calcium deposits (Figure 5B), especially Ga10. Mineralization in Ga10 was almost 300% higher than in Ti and was also the only one sample to improve mineralization obtained by Ti–Ca, by 139%.

### 3.6 The incorporation of Ga promotes the osteogenic differentiation

**FIGURE 6:**
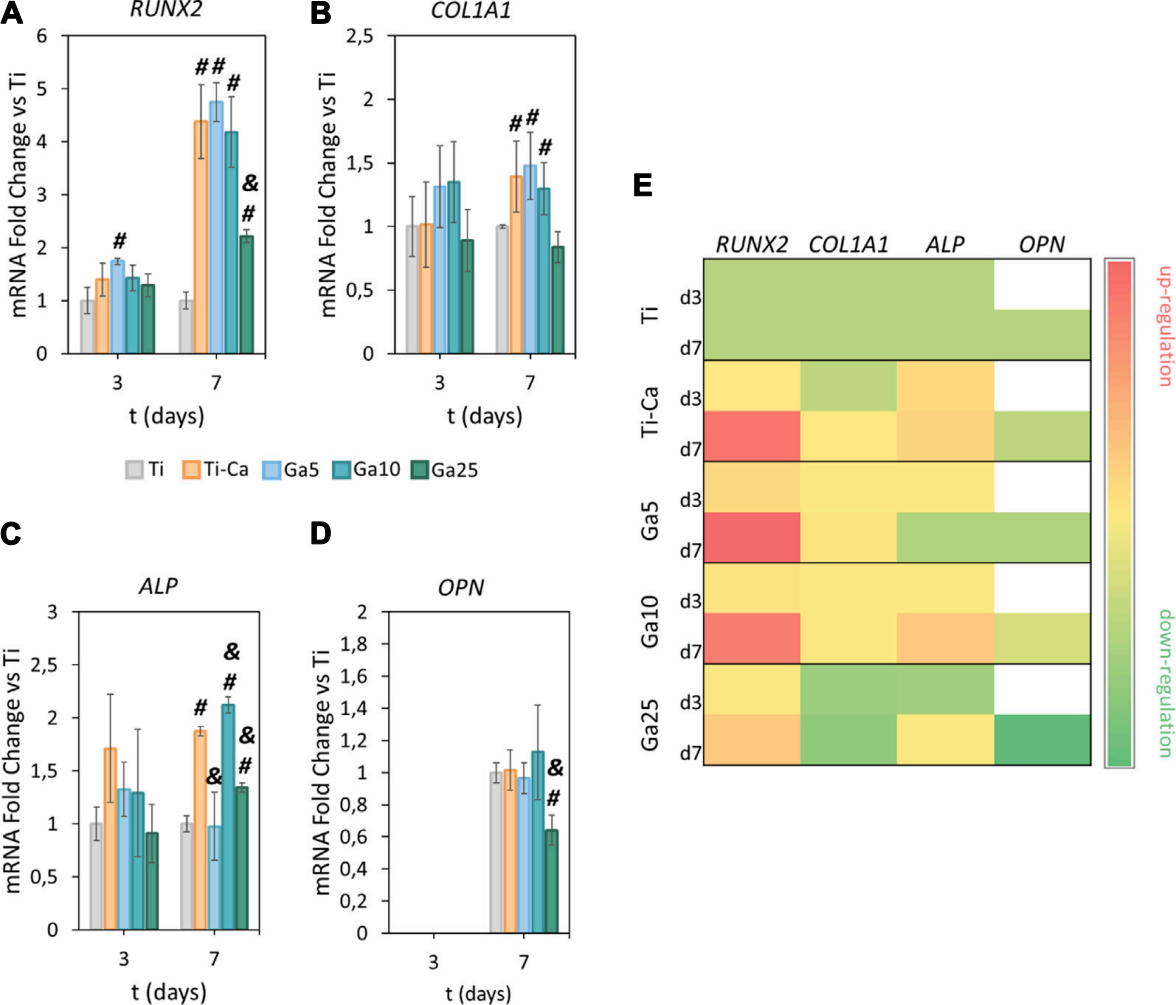
Effects of Ga-containing surfaces in osteogenic expression. RT–PCR analyses of **(A)** RUNX2, **(B)** COL1A1, **(C)** ALP, and **(D)** OPN for hMSCs on days 3 and 7. **(E)** Heatmap of osteogenic gene expression (d3 = day 3, d7 = day 7, red = upregulation, yellow = no changes, and green = downregulation). The results were normalized to housekeeping β-actin and were represented as relative fold change to Ti at each time point. Each condition was replicated in triplets (n = 3). # represents statistically significant differences between treated samples and Ti. & represents statistically significant differences between treated samples and Ti–Ca for each time point (*p*-value <0.05).

RUNX2 was strongly upregulated by the treated samples on day 7 (Figure 6A). Ti–Ca, Ga5, and Ga10 were the samples with the highest upregulation of this gene. Even though the expression of RUNX2 was significantly higher in Ga25 than that in Ti, it was drastically lower than in Ti–Ca and the other Ga-conditions. Concerning COL1A1 (Figure 6B), no significant differences were found on day 3, but Ti–Ca, Ga5, and Ga10 upregulated its expression on day 7. In the case of ALP (Figure 6C), no statistically significant differences were found on day 3, but its expression in Ti–Ca and Ga10 was strongly upregulated on day 7. Ga10 was the condition that most upregulated ALP expression; however, in Ga5 and Ga25, it was similar to Ti. Regarding OPN (Figure 6D), the expression was the same in Ti, Ti–Ca, Ga5, and Ga10; however, in the case of Ga25, it was lower. Overall, Ga10 was the condition that showed a better enhancement of osteogenic expression with an upregulation of RUNX2, COL1A1, and ALP with respect to Ti (Figure 6E).

## 4 Discussion

In this work, we reported a titanium implant subjected to gallium-doped thermochemical treatment capable of inhibiting osteoclastogenesis and inducing osteoblast differentiation. Ga-containing calcium titanate and gallium titanate layer were effectively formed on the surface of titanium as a consequence of the treatment. The presence of this structure was confirmed by Raman spectroscopy (Figures 1A,B), which showed the expected bands in agreement with the literature (Yamaguchi et al., 2017; Rodríguez-Contreras et al., 2020). Moreover, it was found that the presence of rutile and anatase and titanates was usually found in this kind of thermal treatment (Kizuki et al., 2010; Challagulla et al., 2017). Regarding Ga^3+^ release (Figure 1C), all samples showed a quick release in the first hours and Ga10 reached the highest amount of Ga^3+^ released after 14 days.

Interestingly, while the Ga^3+^ release from Ga10 is higher than Ga5, a slower release was found in the samples subjected to the highest concentration of Ga(NO_3_)_3_, Ga25. Kokubo and Yamaguchi (2016) proved that the release of Ca^2+^ from the calcium titanate was significantly lower due to its compact structure. For this reason, a water immersion step was added after the thermochemical treatment (Kizuki et al., 2010). As a result, Ca^2+^ was partially replaced with H_3_O^+^, producing a Ca-deficient calcium titanate that allowed a better ionic exchange with a faster release. The higher concentration of Ga in Ga25 can make the crystallographic structure of Ga-containing calcium titanate tighter and, hence, the release of the Ga^3+^ ions is less efficient than Ga5 or Ga10, according to Kokubo’s finding. Probably, a longer water immersion of Ga25 would lead to a higher release. However, taking into account the good *in vitro* results of Ga10, no modification in the thermochemical treatment was made.

Concerning biological evaluation, Ga-containing samples were capable of inhibiting the formation of the multinucleated cells on its surface (Figure 2A). However, on Ti–Ca and Ga5, large cells were observed under the colonies formed by non-differentiated RAW 264.7, indicating the possibility of further osteoclast formation. Further confirmation of the presence of osteoclasts was carried out by TRAP staining (Yang et al., 2019). The staining confirmed the presence of osteoclasts on TCP, Ti, Ti–Ca, and Ga25 (Figure 2C). The apparition of osteoclast on the surface of Ga25 was expected due to its lower Ga^3+^ release. Regarding osteoclast-related gene expression (Figure 3), Ga10 was the sample that most downregulated NFATc1, TRAP, c-Fos, and MMP9. As it was expected, the lower release of Ga^3+^ in Ga25 showed worse gene expression. The results found here are in agreement with other Ga-doped materials in which Ga reduces osteoclast formation and the expression of osteoclast-related genes (He et al., 2019; Wang et al., 2021).

Few studies have delved into elucidating the mechanism by which Ga reduces osteoclastogenesis. Ga^3+^, as it was mentioned, presents similarity with Fe^3+^, and it has been recently found that iron plays an important role in osteoclast differentiation (Wang et al., 2018). In fact, several kinds of osteoporosis are induced by an abnormality in iron metabolism (Jiang et al., 2022; Liu et al., 2022). The involvement of iron in osteoclastogenesis could arise due to osteoclasts being high energy-demand cells, so pre-osteoclast RANKL-induced cells need a high number of mitochondria, and for this process, iron uptake is promoted and ROS levels are increased (Kim et al., 2017; Li J. et al., 2020; Ni et al., 2021). However, during osteoclastogenesis, iron overload is controlled by iron chelators such as ferritin (Plays et al., 2021), and the ROS production does not lead to cell damage. Nevertheless, a different situation could take place if iron chelators are not available to control the iron overload. In this case, free iron ions in the cytosol could enhance the production of ROS by the Fenton reaction that damages the lipid membrane and causes the death of the cell (Stockwell et al., 2017; Forcina and Dixon, 2019; Li X. et al., 2020; Jiang et al., 2021). Indeed, this kind of cell death, caused by iron overload triggering the production of ROS, was discovered in 2012 and is known as ferroptosis (Dixon et al., 2012). Ga^3+^ has been recently reported as a ferroptosis-inducer agent (Hreusova et al., 2022; Romani et al., 2023). When Ga^3+^ is present during osteoclastogenesis, it could alter the iron metabolism due to its similarity with Fe^3+^, leading to binding with ferritin (Harris and Messori, 2002; Hreusova et al., 2022). Now, Ga^3+^ would act as a “Trojan Horse,” preventing ferritin from binding to Fe^3+^. As a consequence, the iron free from ferritin will accelerate the Fenton reaction (Cai-Hong Zheng et al., 2020) and the ROS levels will be elevated, triggering the cell death by ferroptosis.

**FIGURE 7:**
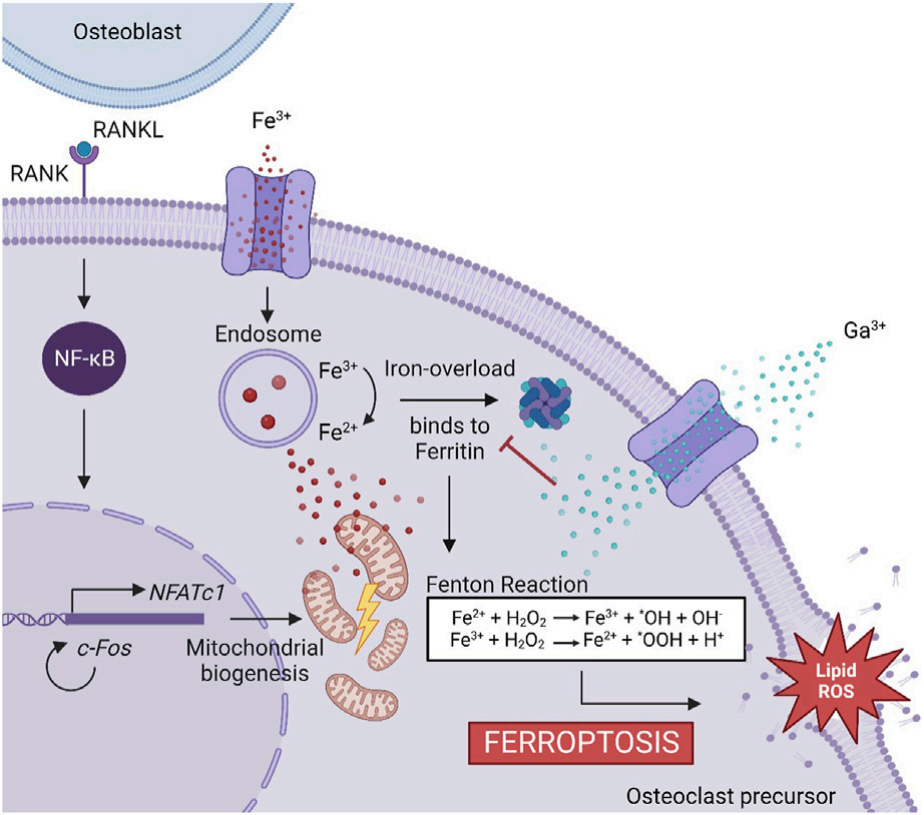
Proposed mechanism of Ga^3+^ to inhibit osteoclastogenesis. The binding of RANKL and RANK induces NF-κB activation and is translocated into the nucleus, promoting the expression of genes involved in osteoclastogenesis. NFATc1 triggers a cascade of cellular events inducing osteoclast recruitment. For this process, mitochondrial biogenesis is needed. The Fe^3+^ uptake is promoted to obtain the energy needed. It produced an iron overload controlled by ferritin. Ga^3+^ is uptaken into the cells and acts as a “Trojan Horse,” preventing ferritin from binding to Fe^3+^. Free iron ions accelerate the Fenton reaction producing ROS, which triggers the cell death by ferroptosis.

The induction of ferroptosis to osteoclast precursors by Ga^3+^ could explain better the results obtained here. A decreased cell population was observed on day 6 in the Ga-containing samples (Figure 2A). Moreover, ferroptosis taking place has sense in the evaluation of osteoclastic genes. When RAW 264.7 cells are induced with RANKL, the osteoclast precursor would activate the expression of osteoclastic-related genes but the process would be interrupted by Ga in the step where iron demand is enhanced. For this reason, NFATc1, TRAP, and c-Fos (Figures 3A–C) were not strongly downregulated by Ga. However, Ga was able to drastically reduce the expression of MMP9 (Figure 3D). This matrix metalloproteinase, which mediates collagen degradation, is produced by the mature osteoclasts (Gu et al., 2014; Guo et al., 2018), and these cells will never start to form. As it was mentioned, few studies have deeply investigated the role of Ga in the process of osteoclastogenesis; for example, Li et al. (2023) found that Ga^3+^ effectively disrupts iron metabolism but the involvement of ferroptosis was not elucidated. We propose ferroptosis as a possible reason (Figure 7); however, further investigations will be required to shed light on this matter.

Since osteoclasts are high energy-demand cells with a large amount of mitochondrial activity (Brown and Breton, 1996; Ishii et al., 2009), it is expected that Ga^3+^ could only interfere in the iron metabolism in these kinds of cells and cells displaying pro-inflammatory events as macrophages (Truong et al., 2023). For this reason, the hMSC behavior in our Ga-containing samples was evaluated. With the introduction of Ga, hMSCs showed an adequate adhesion, area, morphology, and spreading on the surfaces of Ga-containing samples, especially on Ga10 (Figure 4). On day 14, cell proliferation was slightly lower in Ga5 and Ga10 than that in Ga25 and Ti–Ca because Ti–Ca showed a better osteoinductive ability in terms of ALP activity (Figure 5A). Interestingly, low proliferation was related to high osteodifferentiation on Ga-containing calcium titanate, as was found by Rodríguez-Contreras et al. (2020). Therefore, on day 21, when the good osteoinductive properties provided by Ga^3+^ could be observed, cell proliferation in Ga10 and Ga21 was lower. In fact, Ga-containing samples stimulated early differentiation of hMSCs to osteoblasts (Figures 5, 6) showing Ga10 the best performance. These results were in agreement with other studies where Ga promoted osteogenic differentiation (Gómez-Cerezo et al., 2018; Yu et al., 2020). The results of osteogenic expression are in agreement with what found in the work of Piñera-Avellaneda et al. (2023), where RUNX2, ALP, and COL1A1 were upregulated by calcium titanate, which was also found using Ga here. In addition, Ga promoted the calcium deposition after 21 days of culture; therefore, our results suggested that Ga does not have harmful effects in hMSC and promotes the differentiation to osteoblast. In future *in vivo* experiments, Ti subjected to Ga-modified thermochemical treatment is expected to be able to promote adequate osteointegration, as reported in studies using other Ga-based materials (Ravanetti et al., 2016; Wang et al., 2021; Xu et al., 2023)

## 5 Conclusion

Ga-containing calcium titanate inhibits osteoclastogenesis in a dose-dependent manner. The highest Ga^3+^ released from the titanium samples subjected to thermochemical treatment with 10 mM of Ga(NO_3_)_3_ showed the best performance in the osteoclast evaluation assays. Given the results of this work, the idea that Ga^3+^ inhibits osteoclastogenesis by inducing ferroptosis was hypothesized with literature support. Furthermore, Ga-containing samples displayed osteoinductive properties. Therefore, not only is Ga a promising strategy to reduce bone resorption in fractures of patients with osteoporosis or bone metastasis but also Ga could ensure the osteointegration of the implant by promoting bone regeneration.

## Data Availability

The original contributions presented in the study are included in the article/Supplementary Material; further inquiries can be directed to the corresponding author.
